# A Clinical Lecture on Dyspeptic Vertigo

**Published:** 1882-12-20

**Authors:** Alonzo Clark

**Affiliations:** Emeritus Professor of the Principles and Practice of Medicine in the College of Physicians and Surgeons, New York


### A CLINICAL LECTURE ON DYSPEPTIC VERTIGO.

By ALONZO CLARK, M. D.,

**Emeritus Professor of the Principles and Practice of Medicine in the College of Physicians and Surgeons, New York.**

This man is a German, about 50 years of age, and he complains of feeling very dizzy for the past four weeks. He says he went to bed one night feeling all right, and when he awoke in the morning he was unable to get up, and he could not get out of bed for two days; and since that time, though he can get around, yet he has not been able to walk ten feet, he says, without feeling as if he should fall. He has had a pain in his side ever since the war, but he has never been really sick until about four weeks ago. He does not complain of any gastric symptoms, such as belching up of air or passing wind by the bowels, or of any disagreeable sensations after taking food. He has had piles, but they were tied off. He also has peculiar feelings in his hands, and they are weaker than natural, as are also his legs below the knees. But when he closes his eyes and a pin is put between his thumb and fore-finger, he is able to distinguish the head from the point. He is also able to carry his finger to his nose with his eyes closed, without much hesitation, and he can stand steadily with his eyes shut, though not quite as firmly as is natural. He has not been able to work much for the past five or six years, because of the pains in his arms, and he also has a good deal of pain in the upper occipital region of the head. He says, too, that he has had a rheumatism in the legs for two years, and a sensation as if from a thousand stitches begins in his hand and runs up his arm.

There does not seem to be here any pathological change which we can refer to the brain directly, but possibly there may be a congestion of the spinal cord high up where the nerves which go to the arm are given off, and this would account for his peculiar pains in the hands.

A good many years ago Mr. Bird found that nitro-muriatic acid would aid digestion, and he found that two or three drops of the strong acid would relieve dizziness from a disordered stomach, if given at the beginning of a meal. He published a report of his successes, and then I made a trial of this remedy, but I did not administer it just as he directed, but gave it after meals, and in five-drop doses in five table-spoonsful of water, taken through a tube in order to preserve the teeth from the action of the acid, and I often gave ten to fifteen grains of pepsin with it, to aid digestion. I can hardly tell you how many cases of dizziness I have seen cured by this administration. One case I remember was that of a man eminent in politics, who came to me fifteen years ago complaining of dizziness, and I gave him this acid for it. In ten days he came back, and told me that he was now well. He then discontinued the use of the remedy, and in three or four months he came back and asked me for the same prescription, and when he began using it he was cured again.

At another time I was riding in a Third avenue surface car and I noticed that the conductor looked at me very intently, and at last he came up to me and asked if I was not Dr. Clark. I answered, yes. Then he asked if I did not know him, and I said, no. Then he said: "Four weeks ago I came to you because I was dizzy most of the time, and you gave me some acid, and in four or five days I was completely cured." This remedy for dizziness is not in very general use, and it is worthy of being employed far more extensively than it is now, I believe. I am disposed to try this here, though this man does not give us all the symptoms of dyspepsia. He does not give the very common symptom of belching up of wind, but he does complain of a pretty constant pain in the left side, in the region of the stomach. He also speaks of passing balls from his rectum, by which he means, I suppose, sycbalous accumulations of feces, so I would give him laxatives and direct him to eat such things as would aid the action of the bowels. The best of such foods is Graham bread, which is wheat flour ground and not bolted, and it is an excellent laxative. He should eat fruits, too, and perhaps the best are apples, especially if they are baked, and these may be sufficient to keep the bowels in proper condition. These, then, are the two things which I believe he requires. I became very much interested in this class of cases after I had heard of Dr. Bird's plan of treatment, and now I like to see them because I feel that I can give them relief.—*Med. Gazette.*

